# Carbonic anhydrase IV in lizard chemical signals

**DOI:** 10.1038/s41598-023-41012-9

**Published:** 2023-08-29

**Authors:** Marco Mangiacotti, Marco Fumagalli, Claudio Casali, Marco Biggiogera, Federico Forneris, Roberto Sacchi

**Affiliations:** 1 Department of Earth and Environmental Sciences, University of Pavia, Via Taramelli 24, 27100 Pavia, Italy; 2 The Armenise-Harvard Laboratory of Structural Biology, Department of Biology and Biotechnology “L. Spallanzani”, University of Pavia, Via Ferrata 9A, 27100 Pavia, Italy; 3 Laboratory of Cell Biology and Neurobiology, Department of Biology and Biotechnology “L. Spallanzani”, University of Pavia, Via Ferrata 9, 27100 Pavia, Italy

**Keywords:** Chemical ecology, Behavioural ecology

## Abstract

The evolution of chemical signals is subject to environmental constraints. A multicomponent signal may combine semiochemical molecules with supporting compounds able to enhance communication efficacy. Carbonic anhydrases (CAs) are ubiquitous enzymes catalysing the reversible hydration of carbon dioxide, a reaction involved in a variety of physiological processes as it controls the chemical environment of the different tissues or cellular compartments, thus contributing to the overall system homeostasis. CA-IV isoform has been recently identified by mass spectrometry in the femoral gland secretions (FG) of the marine iguana, where it has been hypothesized to contribute to the chemical stability of the signal, by regulating blend pH. Lizards, indeed, use FG to communicate by delivering the waxy secretion on bare substrate, where it is exposed to environmental stressors. Therefore, we expect that some molecules in the mixture may play supporting functions, enhancing the stability of the chemical environment, or even conferring homeostatic properties to the blend. CA-IV may well represent an important candidate to this hypothesized supporting/homeostatic function, and, therefore, we can expect it to be common in FG secretions of other lizard species. To evaluate this prediction and definitely validate CA identity, we analysed FG secretions of eight species of wall lizards (genus *Podarcis*), combining mass spectrometry, immunoblotting, immunocytochemistry, and transmission electron microscopy. We demonstrate CA-IV to actually occur in the FG of seven out of the eight considered species, providing an immunochemistry validation of mass-spectrometry identifications, and localizing the enzyme within the secretion mass. The predicted structure of the identified CA is compatible with the known enzymatic activity of CA-IV, supporting the hypothesis that CA play a signal homeostasis function and opening to new perspective about the role of proteins in vertebrate chemical communication.

## Introduction

Chemical communication is ubiquitous in the animal kingdom, where it shows a variety of forms and designs, each representing an adaptation to different, concomitant evolutionary drivers^1^, not least the environmental background where communication takes place^2–4^. In terrestrial environments, for example, volatility plays a key role, since it determines the range and longevity of a signal, where high volatility corresponds to a rapid diffusion over large distance but also a quick fade^5^. In such context, the optimization of signal efficacy may lead to the evolution of chemical mixtures where semiochemicals, i.e., molecules active in communication^6^, are mixed with supporting compounds, not necessarily involved in signalling. Supporting compounds may work as an “active” chemical matrix able to influence the chemo-physical properties of specific compounds as well as of the whole blend, and eventually allow tuning signal longevity and volatility, according to the environmental conditions, and social functions^4,6–9^. A well-studied example comes from mice (*Mus musculus*), whose urine is enriched by specific proteins (MUPs^10^), which, besides being involved in individual recognition^11^, bind volatile molecules (e.g., 2-*s*-butyl-4,5-dihydrothiazole^12^), thus ensuring their slow release and increasing their longevity^13^. Alternatively, in insects, the array of cuticular hydrocarbons from different classes shows a melting point 15 °C higher to that predicted by the weighted average among the components^14^, thus greatly enhancing the environmental adaptability of the blend^15^. In lizards, cholesterol decreases the volatility of the chemical mixture, and its abundance increases with environmental temperature^16,17^; similarly, α-tocopherol, known for its antioxidant properties^18^, helps stabilizing the secretions in mesic environment^19,20^. Overall, by combining semiochemicals and supporting molecules, multicomponent signals can be adjusted to accommodate the changes in the environmental conditions in a finer way than single-component scents can do^21,22^.

Carbonic Anhydrases (CAs) are a superfamily of metalloenzyme catalysing the reversible hydration of carbon dioxide, an elemental reaction which is involved in a variety of physiological functions (pH regulation, ion transportation, carbon dioxide fixation, calcification, signal transduction;^23–25^). Originally purified from human blood cells^26^, CAs were later found to be common^27^, and many genetic families (α-, β-, γ -, δ-, ζ-, η-) have been recognized^25^. In vertebrates, notably mammals (the best studied group), sixteen α-CA isoforms have been identified, located in different tissues and organs (e.g., blood, muscle, lung, kidney, brain, gut^25,28^), as well in different cellular compartments (cytosol, mitochondria, cytoplasmatic membrane^28^), or even secreted^29–31^.

In 2020, CA-IV was identified by mass spectrometry as one of the most abundant protein in the femoral gland (FG) secretions of the Galapagos’ marine iguana, *Amblyrhynchus cristatus*^32^. The occurrence of this enzyme in FG secretions has been later confirmed in a phylogenetically distant species, the sand lizard, *Lacerta agilis*^33^, and, very recently, also in the Gran Canaria giant lizard, *Gallotia stehlini*^34^. FG secretions are chemical cues used in lizard intra- and interspecific communication^35–40^. Secreted by specialized epidermal glands occurring as two symmetric series along the thighs^41,42^, FG secretions consist of a waxy mixture of lipids and proteins^43–45^. Lipids have been chemically identified and mostly associated to semiochemical function^46–48^. The proteinaceous fraction has been less studied^32, 33,42,49^, and recently characterized only in the two above-mentioned species^32,33^. In both cases, this fraction mainly includes keratins, small serum proteins, and lipid-binding proteins^32,33^, suggesting it can represent the supporting component of the chemical cue^32^, despite previous evidence for additional semiochemical function^8,50,51^.

The CA-IV isoform identified by Tellkamp et al.^32^ is a 35 kDa protein, usually occurring on the extracellular side of the membrane^28^, where it is anchored by a GPI (glycosylphosphatidylinositol) tail^52^. CA-IV is remarkably stable, due to the occurrence of two disulphide linkages^53^, has a C-terminal hydrophobic domain^54^, and keeps its catalytic activity even in its secretory form, i.e., when the C-terminal anchoring domain is removed^55^. The above considerations are fully compatible with a supporting-role in FG secretions, where CA-IV can be directly involved in the chemical homeostasis of the mixture. This hypothesis is made even more intriguing considering that FG secretions are often delivered on bare substrates, like stones, rocks or walls^41,56^, thus potentially experiencing very high temperature, UV exposure, and rapid desiccation^22,57,58^. Consequently, the chemical mixture, and supporting compounds therein, should be able to work also in such extreme conditions.

**Figure 1:**
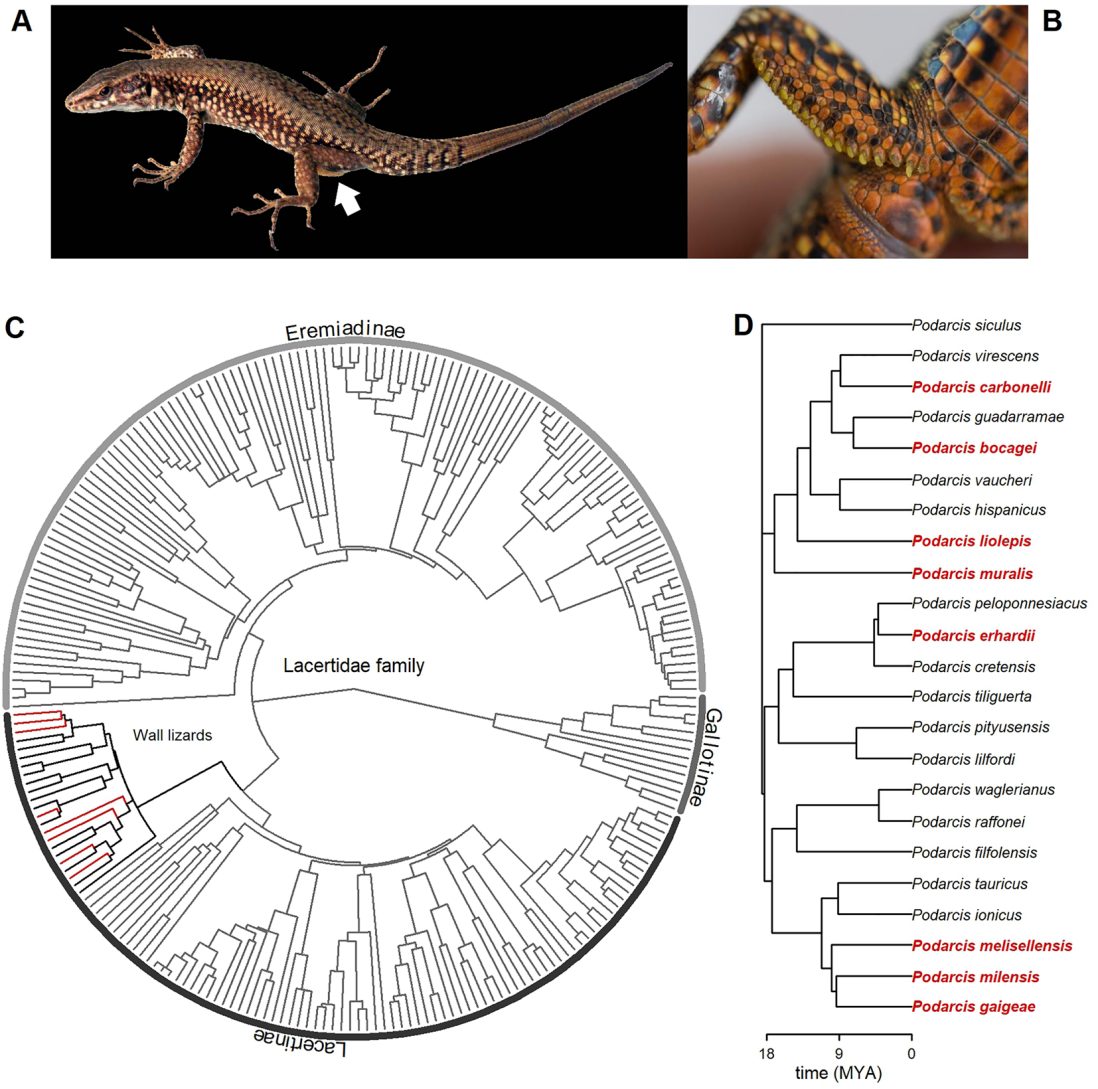
Phylogenetic relation among lizards from the Lacertidae family (modified from^67^) included in the present study. (**A**) photo of a male *Podarcis muralis* with visible femoral gland secretions protruding from the left hindlimb (white arrow). (**B**) particular of the cloacal region of a male *P. muralis* showing the series of femoral pores and yellowish secretions along the thigh—photos by MM. (**C**) phylogenetic tree built on 246 out of 373 currently recognized species belonging to the Lacertidae family^68^; subfamilies are indicated; the wall lizard group (genus *Podarcis*) is in black; the species included in the current study are in red. (**D**) subtree of the wall lizards included in the phylogeny, with the eight species considered in the present study in red; MYA = million years ago. The figure has been generated in R v4.1.2 (https://cran.r-project.org/).

Given its potential role and its wide diffusion among very different taxa^27^, we can expect CA-IV to occur also in the secretions of other lizard species. To test this hypothesis and to confirm, validate and generalize previous findings, we combined mass-spectrometry analysis, immunoblotting techniques, immunocytochemistry, and transmission electron microscopy to detect, identify, and visualize CA-IV in the FG secretions of eight species from the *Podarcis* genus (Fig. 1). The genus includes small-sized lacertid lizards, distributed from Central to Mediterranean Europe, Maghreb, and the Black Sea^59,60^, therefore encompassing a wide range of environmental conditions^61^, and it has been the focus of many studies about chemical communication (e.g.,^37,39,45,49,50, 62–66^). Further, protein electrophoretic profiles of FG secretions for some wall lizard species have been already published^37^, and a putative CA band seems easily identifiable in all, but one, cases, thus offering an ideal case study for an in-depth analysis using a multi-technique approach.

## Results

### Protein isolation from *Podarcis* femoral gland secretions and selection of candidate CA-IV bands

**Figure 2:**
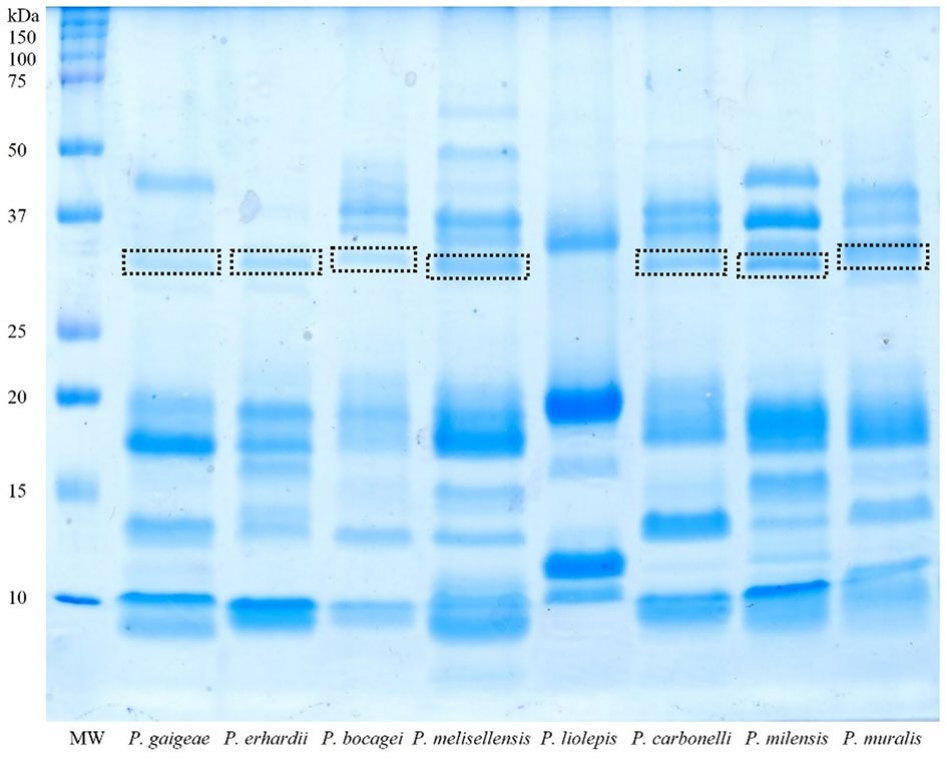
SDS-PAGE analysis of femoral gland secretions belonging to the eight species of *Podarcis* genus under study. The hypothetical band of carbonic anhydrase IV (CA-IV) is indicated by a dotted rectangle; MW indicates the ladder lane, bearing the reference molecular weights. The original gel image is available as supplementary material (Fig. S2).

We successfully managed to extract protein fractions from the femoral gland secretions of all the eight different *Podarcis* species (Table S1). Sodium dodecyl sulphate–polyacrylamide gel electrophoresis (SDS-PAGE) analysis of these samples showed very similar patterns. Their comparison revealed, as expected^37^, limited yet significant qualitative and quantitative differences between the samples (Fig. 2). In particular, we focused on a consistently present protein band found in all samples except in *P. liolepis* (Fig. 2). This protein band had a molecular weight of about 30–35 kDa, which we hypothesized could correspond to CA-IV. The bands of interest were then carefully excised from the gel, destained, digested with trypsin, and resulting peptides were submitted to mass spectrometry.

### Mass spectrometry analysis identifies a CA-IV-like protein in *Podarcis* femoral gland secretions

**Figure 3:**
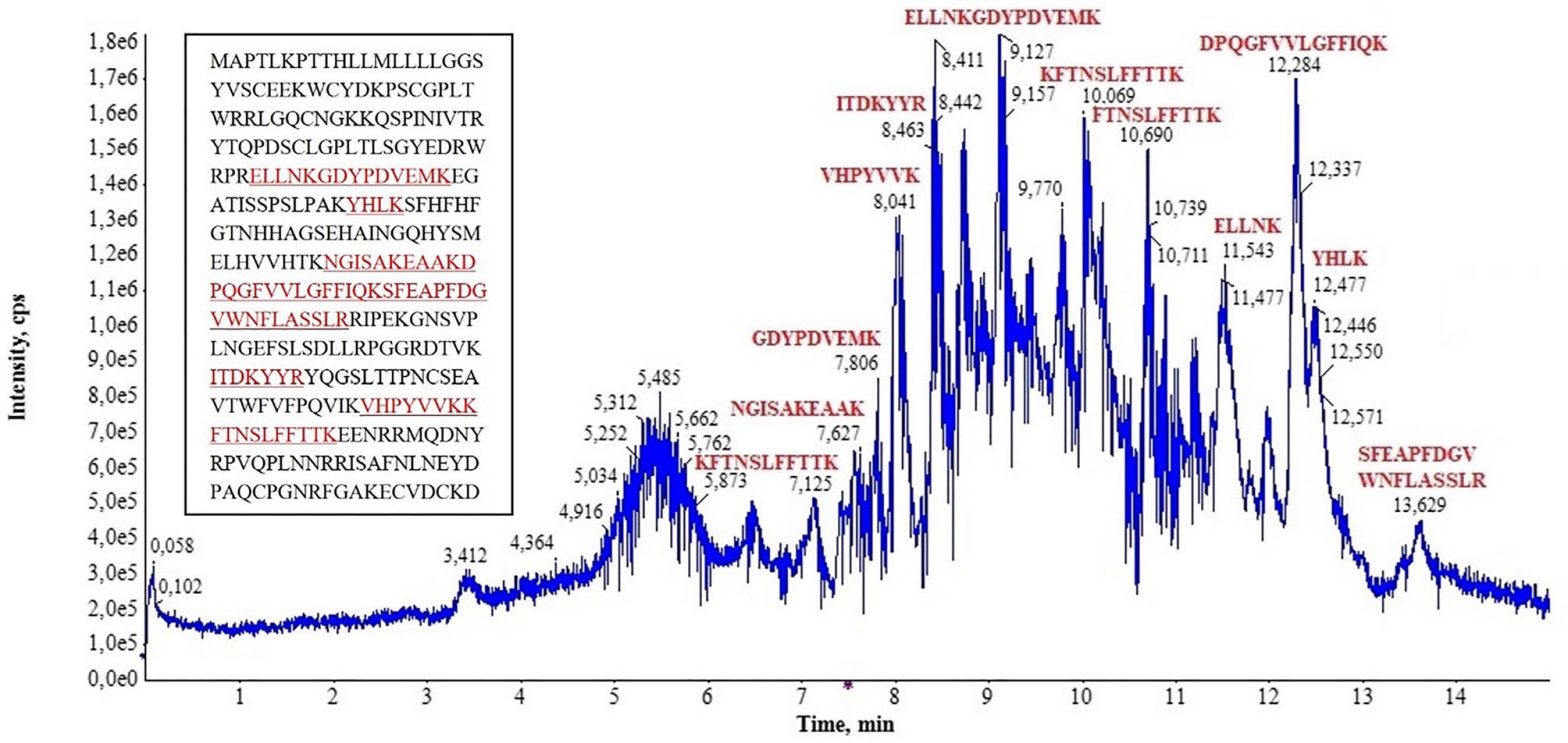
Identification of CA-IV-like protein using mass spectrometry. The chromatogram shows the mass-spectrometry peak profile obtained from tryptic digestion of the sample, with indication of the identified peptides corresponding to each peak. The inset reports the peptide sequence coverage (underlined) on the entire protein sequence.

Mass spectrometry (MS) peaks associated to trypsin-digested polypeptides were searched against the SwissProt and the proteome of *P. muralis* (NCBI txid64176), as extrapolated from its reference proteome as found on the NCBI database. Peptide sequences from *P. muralis* CA-IV-like protein (NCBI reference sequence XP_028560944.1), but not CA-IV (NCBI reference sequence XP_028564309.1) were consistently detected as almost unique hits in all samples analysed (Fig. 3). Despite the lack of available proteomes for other *Podarcis* species, our approach allowed the identification of peptides from CA-IV-like protein sequences in all analysed samples, suggesting a remarkable similarity amongst *Podarcis* species. A detailed overview of MS data, including percent of sequence coverage, number of peptides identified and score percentage of each protein identified in each sample is reported in Table 1.

**Table 1:** Mass spectrometry identification data of carbonic anhydrase IV (CA-IV) in the analysed samples.

Species	Accession	Mass	Score (%)	Coverage (%)	Query matched	Description	Peptides
*P. muralis*	XP_028560944.1	35.858	99	24.37	12	Carbonic anhydrase 4- like [Podarcis muralis]	YHLK
							ELLNK
							ITDKYYR
							VHPYVVK
							KFTNSLFFTTK
							NGISAKEAAK
							GDYPDVEMK
							FTNSLFFTTK
							KFTNSLFFTTK
							DPQGFVVLGFFIQK
							ELLNKGDYPDVEMK
							SFEAPFDGVWNFLASSLR
*P. milensis*	XP_028560944.1	35.858	99	22.78	9	Carbonic anhydrase 4- like [Podarcis muralis]	YHLK
							VHPYVVK
							ITDKYYR
							GDYPDVEMK
							ELLNKGDYPDVEMK
							FTNSLFFTTK
							EAAKDPQGFVVLGFFIQK
							FTNSLFFTTK
							SFEAPFDGVWNFLASSLR
*P. carbonelli*	XP_028560944.1	35.858	99	10.13	7	Carbonic anhydrase 4- like [Podarcis muralis]	YHLK
							ELLNK
							VHPYVVK
							KFTNSLFFTTK
							GDYPDVEMK
							ELLNKGDYPDVEMK
							FTNSLFFTTK
*P. melisellensis*	XP_028560944.1	35.858	99	15.82	7	Carbonic anhydrase 4- like [Podarcis muralis]	YHLK
							VHPYVVK
							KFTNSLFFTTK
							ITDKYYR
							GDYPDVEMK
							FTNSLFFTTK
							SFEAPFDGVWNFLASSLR
*P. bocagei*	XP_028560944.1	35.858	98	5.70	3	Carbonic anhydrase 4- like [Podarcis muralis]	YHLK
							VHPYVVK
							FTNSLFFTTK
*P. erhardii*	XP_028560944.1	35.858	99	19.62	8	Carbonic anhydrase 4- like [Podarcis muralis]	YHLK
							VHPYVVK
							KFTNSLFFTTK
							GDYPDVEMK
							ELLNKGDYPDVEMK
							MQDNYRPVQPLNNR
							FTNSLFFTTK
							EAAKDPQGFVVLGFFIQK
*P. gaigeae*	XP_028560944.1	35.858	99	18.04	9	Carbonic anhydrase 4- like [Podarcis muralis]	YHLK
							RIPEK
							VHPYVVK
							KFTNSLFFTTK
							QSPINIVTR
							GDYPDVEMK
							ELLNKGDYPDVEMK
							MQDNYRPVQPLNNR
							FTNSLFFTTK

Accession: NCBI accession number; Mass, Predicted mass; Score, Peaks overall matching score; Coverage, Percent protein sequence covered by the identified peptides; query matched, Number of identified peptides matching the same protein sequence; description, NCBI description for the accessed entry; Peptides, Identified peptide sequences.

### Western blotting confirms CA-IV in the femoral gland secretions of *Podarcis* species

**Figure 4:**
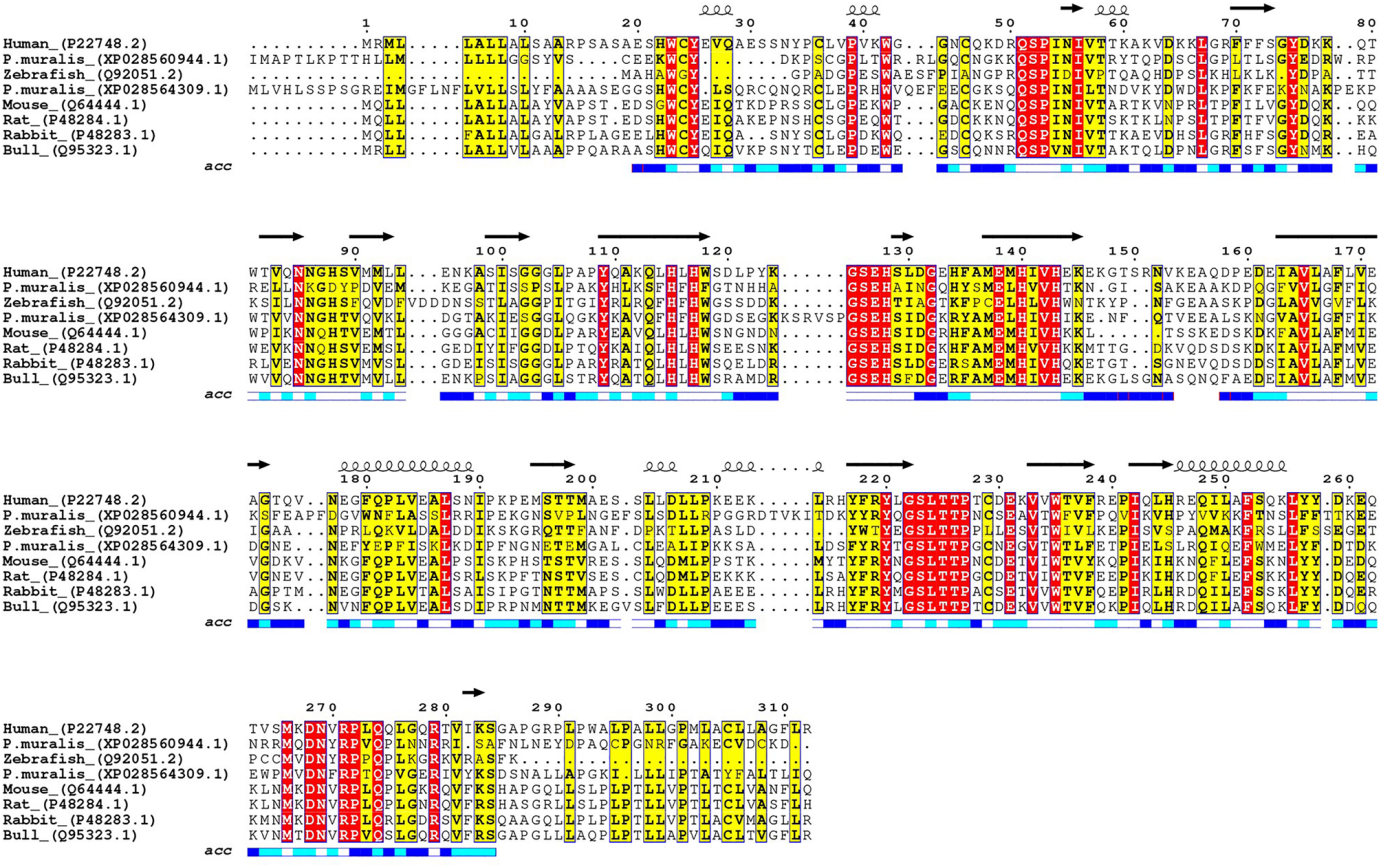
Sequence alignment of CA-IV and CA-IV-like proteins from various vertebrate species. The choice of the species for the alignment was based on the highest scores obtained from NCBI BLAST using the protein sequence of *P. muralis* CA-IV-like protein (XP028560944.1) as search query. The sequence of annotated *P. muralis* CA-IV protein (XP028564309.1) is also included for comparative purposes, although this polypeptide was not identified in the MS experiments. Secondary structure elements (depicted above the alignment) and solvent-accessible residues (acc, below the alignment, with residues in dark blue indicating solvent-exposed residues, in light blue partially exposed residues, and in white residues buried within the globular protein fold) have been computed based on the available crystal structure of human CA-IV (PDB ID 5IPZ;^69^).

**Figure 5:**
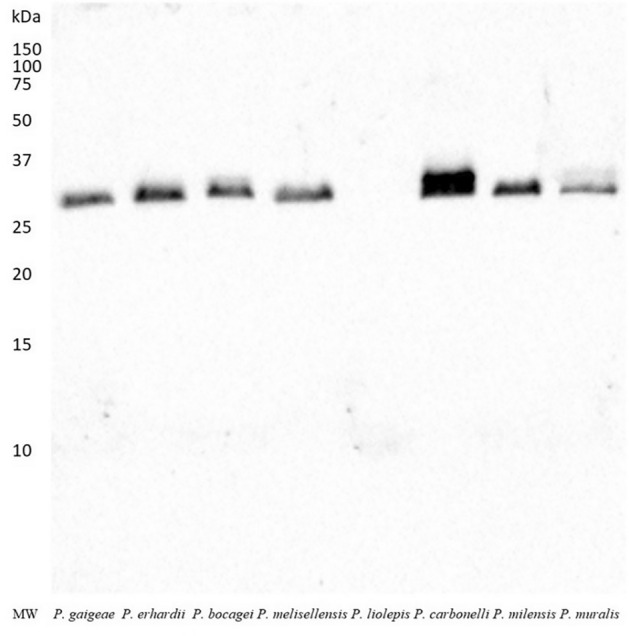
Immunoblotting of proteins from femoral gland secretions. PVDF membrane was incubated with a rabbit polyclonal antibody against vertebrate CA-IV, followed by an anti-rabbit antibody. The only protein band obtained can be associated to CA-IV. The original western blot image is available as supplementary material (Fig. S3).

To further validate the identification of the putative CA-IV-like protein as obtained by mass spectrometry, we opted for western blot analysis of the protein samples. In absence of a *Podarcis*-specific CA-IV antibody, we reasoned on the high degree of sequence conservation between CA-IV proteins of different species (Fig. 4) and opted for using a commercial antibody against vertebrate CA-IV for detection. Samples from each profile of the femoral gland secretions of the various *Podarcis* species were therefore subject run into SDS-PAGE followed by transfer onto a polyvinylidene difluoride (PVDF) membrane and incubation with a primary rabbit polyclonal antibody against vertebrate CA-IV, followed by a polyclonal goat anti-rabbit antibody. A single protein band was consistently revealed by western blotting exactly in the same position as the band used for MS detection (Fig. 5). This result was further confirmed by the absence of bands in the lane corresponding to *P. liolepis*, as already observed in its protein pattern of the SDS-PAGE analysis.

### Ultrastructural analysis localizes CA-IV in femoral gland secretions

**Figure 6:**
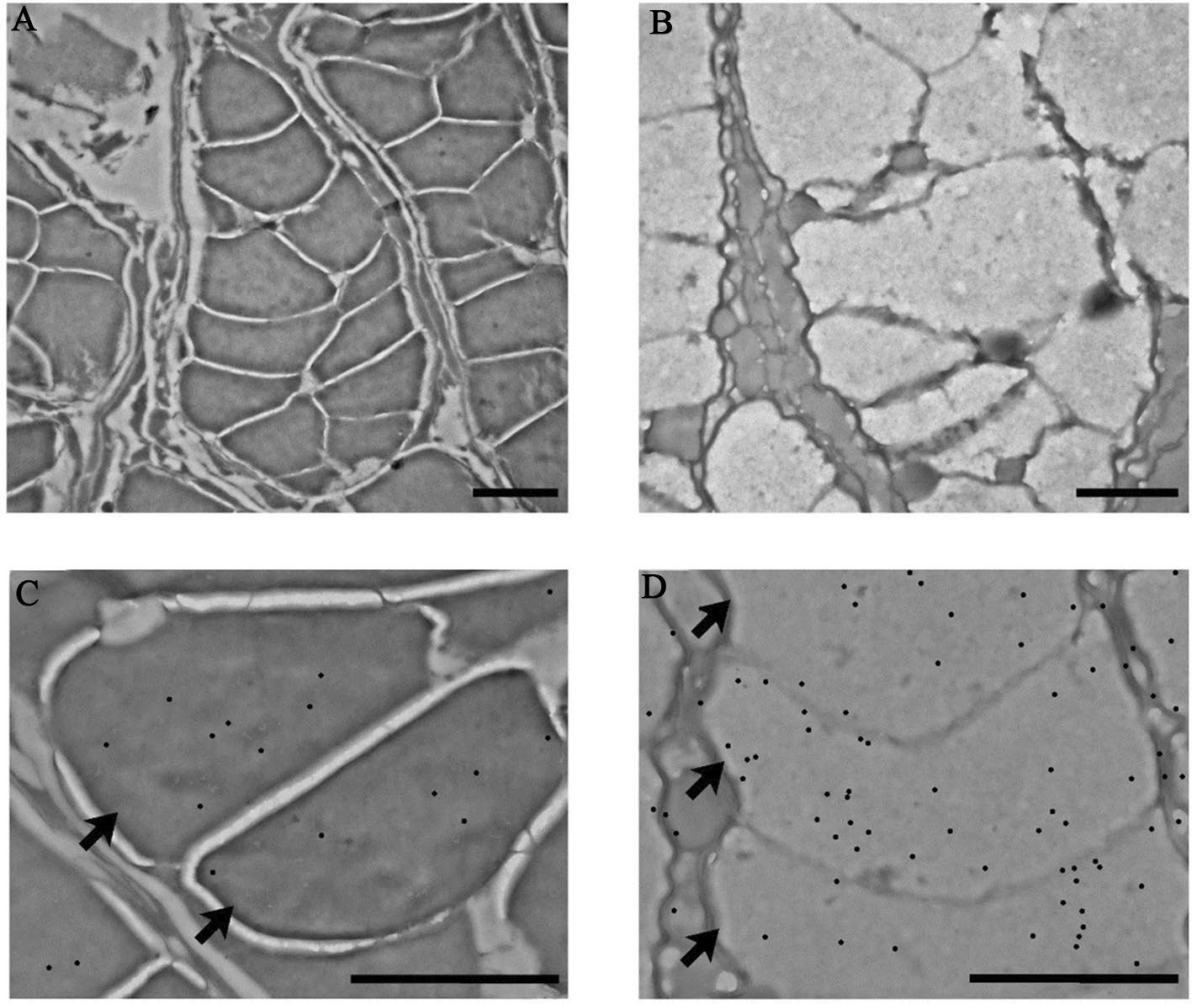
Transmission electron microscopy micrographs of sections of *Podarcis muralis* femoral gland secretions, freshly collected after they were secreted. TEM analyses allowed the identification of compartmentalized structures (**A**, **B**; arrows in **C**, **D**), with different lobes from the same secretion showing various degrees of electron-density, resulting in an overall darker (**A**, **C**) or lighter (**B**, **D**) stain. Black dots (**C**, **D**) indicate colloidal gold particles (post-processed for image readability) resulted from immunolabeling directed against CA-IV, which detected the enzyme in the femoral gland secretions. We report a lower presence of CA-IV in denser secretions (**C**). Scale bar: 2 µm.

Immunocytochemestry (ICC) was performed to detect CA-IV at Transmission Electron Microscopy (TEM). Immunolabeling revealed extensive presence of the enzyme in the femoral gland secretions, identified as the gold particles (Fig. 6). Moreover, the ultrastructural investigation allowed us to notice a degree of variability in the secretions as they are characterized by different levels of electron-density amidst the same sample. Interestingly, we found a correlation between this parameter and the detection of CA-IV immunolabeling. Indeed, darker femoral gland secretions, i.e., the less electron-dense ones, showed higher abundancy of CA-IV (Fig. 6C–D).

## Discussion

The efficacy of chemical communication in terrestrial environment can take advantage from using a multicomponent design^21,22^, where semiochemicals and supporting compounds are combined to confer adaptive and modulable properties to the signal. CA-IV has been potentially identified as the first supporting molecule example in the FG secretions of two lizard species^32,33^. By combining different approaches and techniques, we generalized this finding to seven more lizard species, not only confirming CA-IV occurrence, but also providing the first independent validation of its identity. Notably, (1) the SDS-PAGE profile of all the considered species, except for *Podarcis liolepis*, showed a well-definite band in the predicted molecular range for CA-IV (Fig. 2); (2) MS analysis confirmed all these proteins to match almost uniquely the CA-IV-like sequence of *P. muralis* reference proteome (Table 1; Fig. 3); (3) WB validated CA-IV-like identification only in the species bearing the CA-IV band in SDS-PAGE (Fig. 5); finally, (4) ICC localized CA-IV-like proteins within the secretions (Fig. 6).

**Figure 7:**
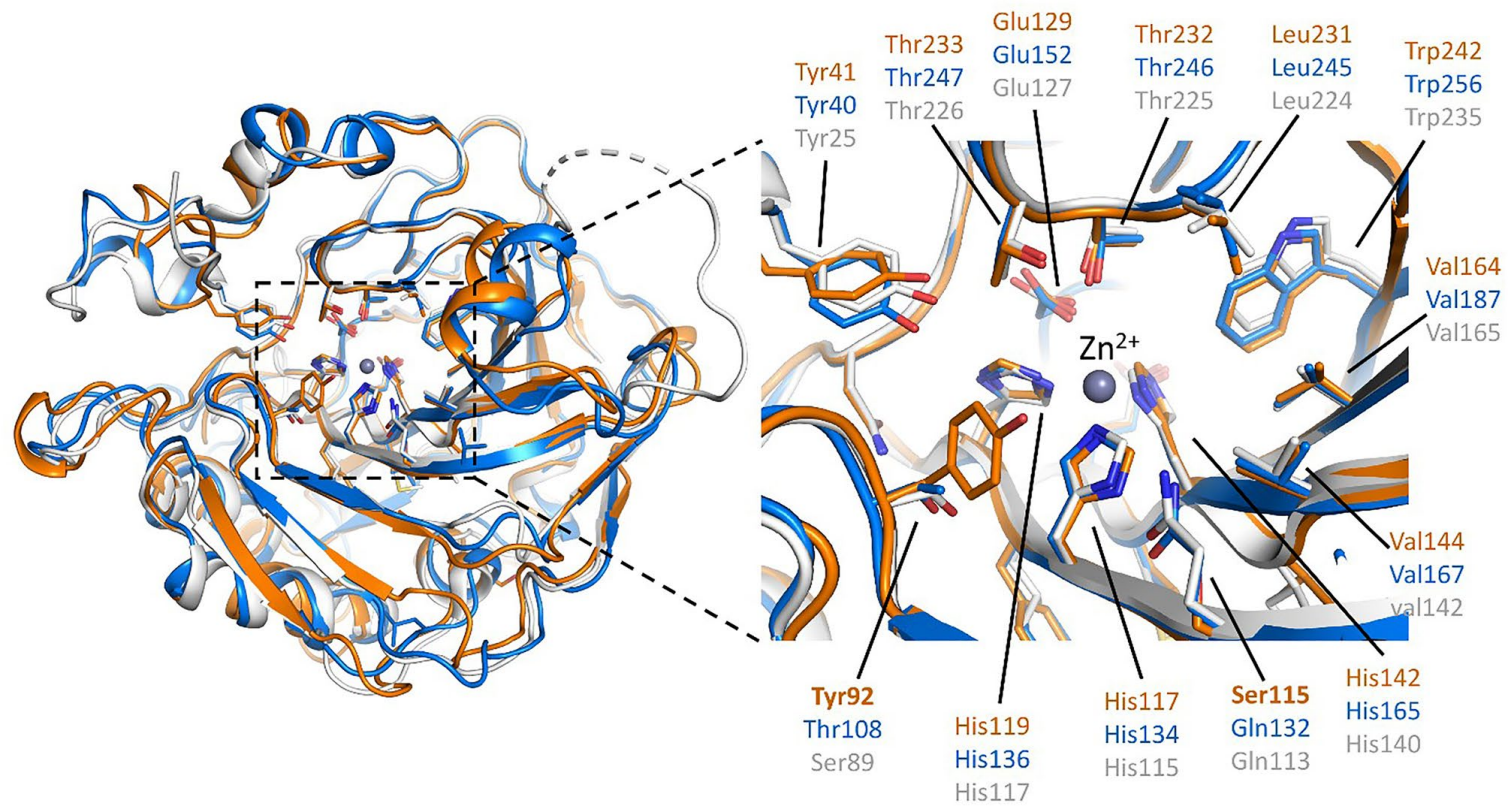
Comparative computational modelling of *P. muralis* CA-IV (blue), CA-IV-like (orange) with the experimental crystal structure of human CA-IV (PDB ID 5IPZ, white). Overall (left panel), the Alphafold2 predictions show remarkable structural resemblance with the human structure. The catalytic site (right panel) highlights a network of highly conserved amino acids, compatible with carbonic anhydrase activity for both *Podarcis* CA-IV and CA-IV-like isoforms. Amino acid side chains showing differences between *P. muralis* CA-IV and CA-IV-like are shown in bold. Figures were generated using the software PyMOL v2.4 (https://pymol.org/2/) and assembled using R v4.1.2 (https://cran.r-project.org/).

Besides confirming and validating the occurrence of CA-IV in lizards’ FG secretions, our results provide circumstantial evidence of the hypothesis that CA-IV plays a supporting function. Firstly, all CAs seem to be rather similar with each other, since both MS and WB were able to identify them basing on the information from the reference proteome of a single species, *P. muralis*, the only available at the time of the analysis for the whole genus. This agrees with the supporting function hypothesized for CA. Indeed, although some species-specific patterns have been recognized in the FG protein profiles of lacertid lizards^37^, we should expect supporting compounds not to follow such pattern, since they should mainly respond to the signalling context (e.g., the environmental conditions;^58,66^) rather than to the conveyed information, such as species identity^70^. Secondly, the computational structural predictions based on Alphafold2 pipelines^71^ highlighted that *P. muralis* CA-IV-like and CA-IV share the same folding topology and their catalytic sites are compatible with binding of Zn^2+^, essential cofactor for carbonic anhydrase activity (Fig. 7;^25,53^). The main differences within the catalytic site were associated to *Podarcis* CA-IV-like Tyr91 replacing CA-IV Thr108 at a site critical for substrate processing (Fig. 7). Notably, this site hosted a conserved small polar side chain (i.e., either Ser or Thr) in most vertebrate CA-IV enzymes (Fig. 4). Collectively, these observations suggest that *Podarcis* CA-IV-like could act as a carbonic anhydrase enzyme similar to its annotated CA-IV counterpart, but with different substrate specificity in reason of its localization in the FG secretions. Thirdly, ICC showed CA to be scattered within the secretions, and not clustered along the boundaries of the compartmentalized ultrastructure (Fig. 6). This may suggest CA to be spread in the mixture, ensuring a homogeneously spatial distribution also of its enzymatic action, which, in turn, agrees with its potential homeostatic function (e.g., pH-regulation;^28^). Further, it also implies CA-IV not to be bound to membrane structures, as expected by the analogy with mammal CA-IV^28,72^: therefore, the identified CA-IV-like protein represents a secreted form, i.e., without the anchoring GPI group^52,54^, which keeps its catalytic activity^55^. Lastly, despite widespread within the secretion mass, CA concentration seemed to be inversely related to the electron-density of the mixture (Fig. 6C–D). This may mean that: (i) secretions are not completely homogeneous (Fig. 6A–B), showing some degree of compositional variation at local scale, as already hypothesized for worm lizards^73,74^; (ii) CA concentration follows such variation, suggesting it can be tuned according to the local composition of the mixture, which is in line with a supporting function.

CAs catalyse bicarbonate dehydration/carbon dioxide hydration, a basic reaction which may directly and indirectly affect a number of processes^23–25^. Following Tellkamp et al.^32^ and Ibanez et al.^33^, we may speculate CA in FG secretions to act on pH regulation, a pivotal parameter of the blend that, in turn, may influence the stability, volatility, and activity of other compounds, be they semiochemicals or supporting molecules, lipids^75,76^ or proteins^77,78^. This way, CA may confer dynamic properties to the mixture, making it able to respond to the variation of the ambient conditions (e.g., temperature, humidity, …), and contributing to the system homeostasis of the FG secretions, similarly to what happens for other isoforms in mammal secretions (milk, tear fluid, saliva;^29,31,79,80^). Recently, CA abundance in FG secretions of lacertid lizards has been found to correlate with that of provitamin D_3_^34^, a molecule known for its semiochemical role in lizard communication^81^. This is in line with what we could expect if CA helped stabilizing other, more labile molecules. Of course, we cannot rule out other functions, and targeted studies are required to verify the above hypothesis.

Finally, WB failed to detect CA-IV in *P. liolepis* (Fig. 5). Although this outcome validates the specificity of the immunolabeling and reinforces the reliability of the results for the other species, it also opens the question about the lack of CA-IV in the FG secretions of this species. We can reasonably exclude it is a false-negative, since the SDS-PAGE profile (Fig. 2) is almost identical to the average profile of the species (see Figs. 1 and 2 in^37^), where the CA-IV band is missing. The absence of CA-IV from the secretions may mean this enzyme not to be pivotal in guaranteeing the efficacy of the chemical signal in this species, although *P. liolepis* uses FG secretions in intraspecific-communication^82^. Nor we can exclude that the missing piece falls within the intraspecific variability of the species (our samples come from a high-elevation population; Table S1). Such pattern has been documented for the lipophilic fraction of the secretions in this species, where populations living at different altitudes showed unique compounds^83^.

In conclusion, we established that CA-IV certainly occurs in the FG secretions of *Podarcis* lizards, generalizing and validating previous findings from two non-congeneric species. This CA isoform is almost homogenously spread amidst the blend, where it may contribute to the system homeostasis, exemplifying what we defined a supporting function. Further studies are necessary to confirm the latter point, and to assess whether CA-IV holds its enzymatic activity within the waxy mixture, notably in view of the potentially extreme environmental conditions the cues have to experience. Moreover, other proteins may be involved in supporting functions and our first assessment opens up to future research on the subject.

## Methods

### Samples collection

For the analysis, we used samples of femoral gland secretions collected from two adult males of each of the following eight species (Fig. 1): *Podarcis bocagei*, *P. carbonelli*, *P. erhardii*, *P. liolepis*, *P. melisellensis*, *P. milensis*, *P. muralis*. The choice to focus on wall lizards (genus *Podarcis*) depended on a combination of reasons. Notably: (1) a preliminary comparison of the electrophoretic profiles available for 36 lacertid lizards^37^ showed that a potential CA band (visible as a peak just before or corresponding to the 40 kDa tick mark; see Figs. 1 and 2 in^37^) seemed to occur in almost all the eleven mapped species, therefore making easier to target and analyse the bands; (2) a single species (*P. liolepis*) did not show such a peak, providing a potential negative control to the experiment; (3) despite sharing many ecological traits, the geographic distribution of the chosen taxonomic group encompasses quite different environments, and spatial extent^59–61^; (4) we were able to collect original samples for eight of these species from different research groups. Samples were collected between 2007 and 2015 (Table S1; Fig. S1), during the breeding season (maximum of glandular activity;^39^). Lizards were captured by noosing^84^ and epidermal gland secretion were collected by gently pressing around the femoral pores while wearing fresh nitrile gloves: the procedure was not invasive and did not cause damage to any animal tissues^64^. All lizards were released healthy at capture points after sample collection. Samples of femoral gland secretions were stored in glass vials fitted with Teflon-lined stoppers, and kept at − 20 °C until analysis. Capture and handling procedures were performed in accordance with the National relevant guidelines and regulation: Croatia (UP/I-612-07/14-48/111 & UP/I-612-07/14-48/33); Italy (PNM-2015-0010423); Greece: all species were collected in accordance with the Hellenic National Legislation (Presidential Decree 67/81); Spain: captures of lizards and sampling procedures were performed under different licenses for the Environmental Agencies of the different Regional Governments of Spain where lizards were studied. This study followed the recommendations reported in the ARRIVE guidelines^85,86^ and was approved by University of Antwerp (Belgium) animal welfare standards and protocols (ECD 2014-32).

### Protein extraction and quantitation

All chemicals were > 98% purity and purchased from VWR unless specified. Protein from FG secretions were extracted following^39,49^. Briefly: secretions were defatted by adding 200 μL of n-hexane to samples (an average of 1–2 mg of dense waxy material) and incubating at room temperature for 2 h. Afterwards, the samples were centrifuged (14,000 rpm for 10 min), and proteins were enriched within the pellet fraction. The procedure was repeated three times and proteins were finally air-dried. Protein pellets were then dissolved in 200 μL of 10 mM PBS buffer pH 7.4, containing 137 mM NaCl and 2.7 mM KCl. The total protein concentration was determined by applying the Bicinchoninic Acid (BCA) assay using a calibration curve obtained with bovine serum albumin (BSA) at concentrations between 5 and 25 μg/mL.

### Sodium dodecyl sulphate–polyacrylamide gel electrophoresis (SDS-PAGE)

Aliquots containing a maximum of 10 μg of proteins for each sample were added to 10 μL of loading buffer solution (50 mM Tris–HCl pH 6.8, 2% sodium dodecyl sulphate, 0.1% bromophenol blue, and 10% glycerol). Prepared samples were denatured by incubating at 95 °C for five minutes. Electrophoretic runs were performed in a discontinuous mode (5% stacking gel and 15% running gel) by applying a constant voltage of 180 V for 2 h. Gels were stained with a 0.12% (*w/v*) Coomassie Blue G-250 solution, containing 40% ethanol (*v/v*) and 10% (*v/v*) acetic acid; gels were destained using a solution of 5% (*v/v*) acetic acid. After running a first gel with both samples for each species, we chose the sample with the best electrophoretic profile (Figs. 2 and S2), notably in the CA band region. This set of eight samples (one for each species) was eventually used for all subsequent analyses.

### In situ enzymatic digestion

The selected bands were excised from the gel, placed into 1.5 mL tubes and broken into small pieces. This material was then washed with aliquots (200 μL) of 100 mM ammonium bicarbonate buffer pH 7.8, 50% acetonitrile (ACN) until complete destaining. Gels were dehydrated by addition of ACN (100 μL). After removal of the organic solvent by air-drying, reduction was performed by addition of 50 μL of 10 mM dithiothreitol (DTT) solution (30 min at 37 °C). DTT was then replaced with 50 μL of 55 mM iodoacetamide for 45 min at 60 °C. The solution was then removed, and the gel pieces were washed twice with 200 μL of 100 mM ammonium bicarbonate for 10 min. The resulting gel was dehydrated by addition of 200 μL of ACN until the gel pieces became of an opaque-white colour. ACN was finally removed, and gel pieces were air-dried. Gels were rehydrated once again by addition of 75 μL of 100 mM ammonium bicarbonate buffer pH 7.8, containing 20 ng/μL sequencing grade trypsin (Promega) and digestion was performed overnight at 37 °C. Following enzymatic digestion, the resultant peptides were extracted sequentially from gel matrix by a two-step treatment (each step for 15 min, while vortexing) with 100 μL of 50% ACN in water, containing 5% formic acid (FA). The original supernatant and those obtained from sequential extractions were pooled, dried under vacuum, and stored at − 20 °C until mass spectrometry (MS) analysis. At the moment of use, the peptide mixtures were solubilized in 50 μL of water containing 0.1% formic acid (FA) for MS analyses.

### Mass spectrometry (MS) analysis

All analyses were carried out with a LC unit (ExionLC AD, AB Sciex) equipped with a column oven thermostated at 40 °C, an autosampler cooled at 10 °C and a binary gradient pump system. MS instrument consists of a high resolution QTOF mass spectrometer (X500B, AB Sciex) equipped with a Turbo V Ion source and a Twin Sprayer ESI (electrospray ionization) probe, controlled by the OS 2.1 software (AB Sciex). Peptides were separated by reverse phase (RP) HPLC on a Hypersil Gold (Thermo Fisher Scientific) C18 column (150 × 2.1 mm, 3 μm particle size, 175 Å pore size) using a linear gradient (2–50% solvent B in 15 min) in which solvent A consisted of 0.1% aqueous FA and solvent B of ACN containing 0.1% FA. Flow rate was 0.2 mL/min. Mass spectra were generated in positive polarity under constant instrumental conditions: ion spray voltage 4500 V, declustering potential 100 V, curtain gas 30 psi, ion source gas 1 40 psi, ion source gas 2 45 psi, temperature 350 °C, collision energy 10 V. Spectra were acquired the generated data were processed with Peaks studio 4.5 software for protein identification. The mass list was searched against the SwissProt and ad hoc databases.

### Western blotting (WB)

Separated proteins were transferred onto polyvinylidene fluoride (PVDF) membrane with a Trans-Blot Turbo Transfer System (Bio-Rad), using a pre-programmed protocol for mixed molecular weights (1.3 A constant current for 7 min). After 1 h incubation in the blocking solution (5% bovine serum albumin, BSA, in Tris-buffered saline, TBS, buffer) and three additional washes with TBS supplemented with 0,1% Tween (TBST), the membrane was incubated overnight at 4 °C with a primary rabbit polyclonal antibody against vertebrate CA-IV (Proteintech), diluted 1:1000 in 1% BSA in TBST. After washing the membrane three times with TBST, incubation with a horseradish peroxidase (HRP)-conjugated goat anti-rabbit secondary antibody (Proteintech), diluted 1:10,000 with 1% BSA in TBST, was carried out for 1 h at room temperature. The membrane was finally washed three times with TBS and incubated with the Clarity wester ECL detection substrate (Bio-Rad). Immunoblots were acquired with the ChemiDoc MP System (Bio-Rad).

### Bioinformatics analyses

CA-IV (XP_028564309.1) and CA-IV-like (XP_028560944.1) protein sequences were retrieved from the proteome of *P. muralis* (NCBI txid64176) deposited on NCBI. Homologous searches were carried out using NCBI BLAST. Results were narrowed based on the annotations for CA-IV or CA-IV-like proteins. The retrieved sequences were downloaded and subject to multi-sequence alignment using EBI MUSCLE^87^, and the computed alignments were displayed using ESPRIPT3^88^. Structural predictions of *P. muralis* CA-IV and CA-IV-like proteins were generated using Alphafold2 pipelines^71^ through the COSMIC website^89^. Comparisons with the experimental structure of human CA-IV (PDB ID 5IPZ,^69^), superpositions and structural figures were carried out using PyMOL^90^.

### Immunocytochemestry (ICC) and transmission electron microscopy (TEM)

For transmission electron microscopy, femoral gland secretions were fixed by immersion in 4% (*v/v*) paraformaldehyde in PBS for 2 h at 4 °C. After several rinses in PBS, samples were incubated in 0.5 M NH_4_Cl in PBS to block free aldehydic groups, dehydrated in a graded ethanol scale and then embedded in LR White resin polymerized for 24 h at 60 °C. Ultrathin sections (60–80 nm) were cut on a Reichert OM-U3 ultramicrotome, collected on nickel grids coated with a Formvar-carbon layer. For the immunocytochemical analysis, sections were floated on normal goat serum (NGS) diluted 1:100 in PBS for 3 min and then incubated over-night at 4 °C with the primary antibody directed against vertebrate CA-IV (Proteintech) diluted 1:50 in PBS containing 0.05% (*v/v*) Tween20. After rinsing, sections were floated on NGS and then incubated for 30 min at RT with a 12 nm gold-conjugated secondary antibody (Jackson ImmunoResearch Laboratories Inc.) diluted 1:20 in PBS. Then, sections were rinsed, air-dried and stained. In detail, grids were incubated for 10 min at RT in uranyl acetate and then in lead citrate for 2 min at RT.

The specimens were observed with a JEM 1200 EX II (JEOL, Peabody, MA, USA) TEM operating at 100 kV and equipped with a MegaView G2 CCD camera (Olympus OSIS, Tokyo, Japan).

### Supplementary Information

Supplementary Information.

## Data Availability

Raw mass spectrometry data associated with the manuscript are available at: 10.5281/zenodo.8288458.
