# Supplementary material for: Carbonic anhydrase IV in lizard chemical signals
Source: Sci Rep. 2023 Aug 29;13:14164. doi: 10.1038/s41598-023-41012-9 (PMC10465503; doi:10.1038/s41598-023-41012-9)

**Table S1**. List of the species considered in the analysis of the protein from the femoral gland secretions. Geographic coordinates (degrees), elevation (m. a.s.l.), and sampling period are given for each sampling site and species.

| Species | Latitude N | Longitude E | Elevation (m a.s.l) | sampling period |
|---|---|---|---|---|
| *Podarcis bocagei* | 42.74 | -9.08 | 56 | May 2007 |
| *P. carbonelli* | 40.50 | -6.09 | 961 | May 2007 |
| *P. erhardii* | 37.83 | 24.85 | 873 | May 2014 |
| *P. gaigeae* | 38.95 | 24.52 | 93 | May 2014 |
| *P. liolepis* | 42.75 | 1.84 | 1773 | May 2012 |
| *P. melisellensis* | 45.10 | 14.34 | 347 | May 2013 |
| *P. milensis* | 36.69 | 24.44 | 17 | May 2015 |
| *P. muralis* | 45.24 | 9.23 | 78 | May 2011 |

**Fig. S1**. Maps of the sampling localities for each species.

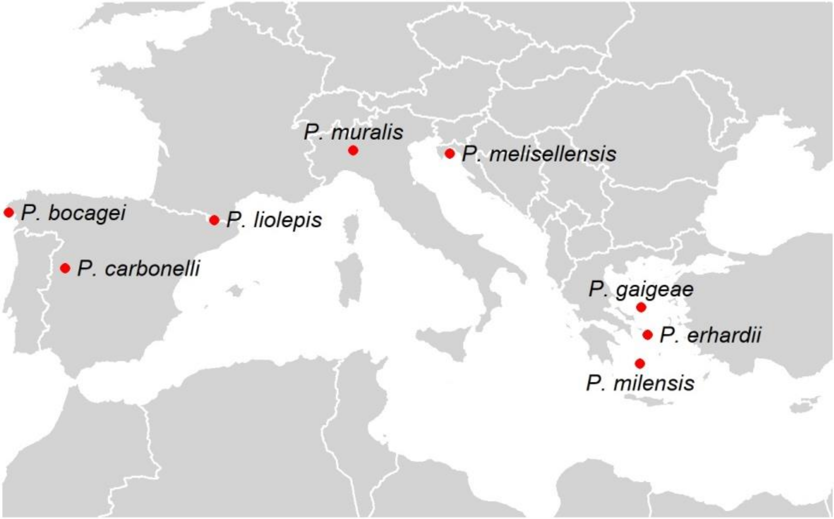

**Fig. S2.** Original image of the SDS-PAGE reported in Fig. 2 before cropping and adding rectangles to denote putative CA-IV bands.

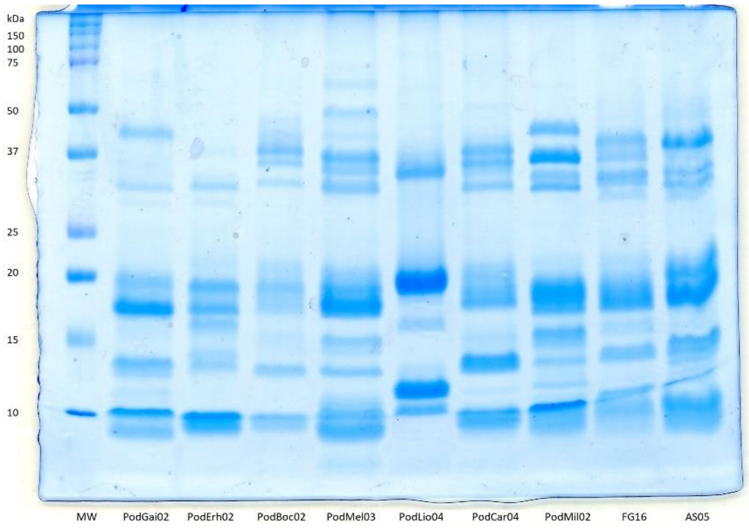


**Fig. S3.** Original image of the western-Blot reported in fig. 5 before cropping and adding molecular weights labels.

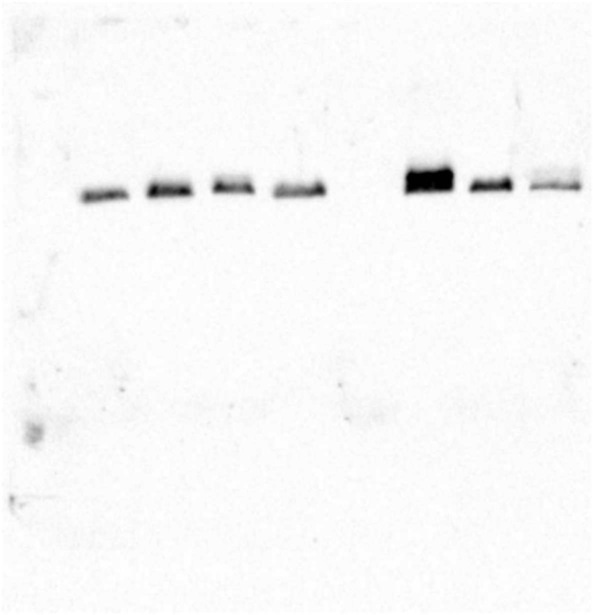

Supplement: Supplementary file 1 — Supplementary Information. [file 41598_2023_41012_MOESM1_ESM.pdf]
